# Long term follow-up of a randomised controlled trial of services for urinary symptoms

**DOI:** 10.1186/1472-6963-11-58

**Published:** 2011-03-14

**Authors:** Kate S Williams, Dawn Coleby, Keith R Abrams, David A Turner, Christine Shaw, R Philip Assassa, Nicola J Cooper, Madeleine MK Donaldson, Catherine W McGrother

**Affiliations:** 1 Department of Health Sciences, University of Leicester, 22-28 Princess Road West, Leicester LE1 6TP, UK; 2 Mid Yorkshire Hospitals, Aberford Road,Wakefield,WF1 4DG, UK; 3 Wessex Institute University of Southampton,Alpha House, Enterprise Rd,Southampton Science Park, Chilworth, Southampton SO16 7NS, UK; 4 University of Glamorgan, UK. Glyntaff Campus,Pontypridd,CF37 1DL, UK

## Abstract

**Background:**

Given the extent and priority of urinary symptoms there is little evidence available to inform service provision in relation to the long term effects of interventions. This study aims to determine the long term (6 year) clinical effectiveness and costs of a new continence nurse led service compared to standard care for urinary symptoms.

**Methods:**

A long term follow-up study of a 2-arm, non-blinded randomised controlled trial that recruited from a community based population between 1998-2000 in Leicestershire and Rutland UK was undertaken. 3746 men and women aged 40 years and over were followed up from the original trial. The continence nurse practitioner (CNP) intervention comprised a continence service provided by specially trained nurses delivering evidence-based interventions using pre-determined care pathways. The standard care (SC) arm comprised access to existing primary care including General Practitioner and continence advisory services in the area. Primary outcome: Improvement in one or more symptom. Secondary outcomes included: a) Leicester Impact scale; b) patient perception of problem; c) number of symptoms alleviated and cost-effectiveness; all were recorded at long term follow-up (average 6 years) post-randomisation.

**Results:**

Overall at long-term follow-up (average 6 years) significantly more individuals in the CNP group (72%) had improved (i.e had fewer symptoms) compared to those in the SC group (67%) (difference of 5% 95% (CI = 0.6 to 9;p = 0.02)).

**Conclusion:**

The differences in outcome between the two randomised groups shown immediately post treatment had decreased by half in terms of symptom improvement at long term follow-up. Although the difference was statistically significant, the clinical significance may not be, although the direction of the difference favoured the new CNP service.

## Background

Urinary symptoms pose a considerable health care burden with 200 million people suffering from incontinence worldwide [1]. In the largest comprehensive study of urinary symptoms in a UK general population, the Leicestershire MRC incontinence study reported that 29% of men and 34% of women aged 40 years or over experience clinically significant storage symptoms with considerable impact on quality of life[2]. This represents a financial burden to the NHS of 1% of its annual budget[3]. Overall prevalence and service needs will continue to grow as the population ages. Consequently it is crucial that effective service interventions with lasting effect that are acceptable to patients are identified.

Given the extent and high priority of this condition there is little evidence available to inform service provision in relation to the long term effects of interventions. The only available evidence to date is a study by O'Brien et al 1991, 1995 [4,5] that assessed the long-term effectiveness of nursing interventions for women with urinary incontinence. This study found that 69% (n = 158) of women who had received treatment by a nurse in the original trial maintained their improvement at 2 year follow-up.

The Leicestershire MRC incontinence programme *'A population laboratory approach to the epidemiology and evaluation of care' *undertook a randomised controlled trial (RCT) of a new continence nurse practitioner (CNP)-led service for urinary symptoms [6], with outcomes at 3 months (immediately post-treatment) and 6 months. The new CNP led service proved to be the most effective with a 10% higher cure rate than standard care (SC) with statistically and clinically significant reductions in urgency, frequency and nocturia as well as incontinence. In addition, quality of life improvements were greater in users of the CNP led service and higher levels of patient satisfaction were achieved. This was the first study to show the effectiveness of nursing services on urinary *storage symptoms *(rather than simply incontinence) and associated QOL. This follow-up study determines the long-term outcomes from this RCT. It is crucial to establish whether the identified differences in outcomes between the standard care (SC) service and the new continence nurse practitioner (CNP) led service were maintained.

## Methods

### Design

This study is a follow-up study of a 2-arm, non-blinded randomised controlled trial that recruited from a community based population in Leicestershire and Rutland between 1998-2000[6]. Follow-up was undertaken in 2006.

### Sample

All individuals were recruited to the original trial with one or more of the following symptoms: *incontinence *several times per month or more, or several times a year plus reported impact[7]; *frequency*, hourly or more, or two hourly plus impact; *nocturia*, three times per night, or twice a night plus impact; or *urgency*, very strong or overwhelming, or strong with impact.

### Randomisation

Randomisation to the original trial was undertaken by household following informed consent at a ratio of 4:1 (CNP:GP). This allocation was necessary in order to ensure sufficient numbers in the nested trials (reported elsewhere) for detrusor overactivity and urodynamic stress incontinence. The statistical programme SAS was used to generate the random allocation sequence and was implemented using sealed envelopes, numbered sequentially.

### Intervention

The participants were randomised to receive either: (i) a continence service provided by specially trained nurses delivering evidence-based interventions using pre-determined care pathways or (ii) standard care which comprised usual access to GP and existing continence services. The duration of the intervention was eight weeks. Full details of study methods, interventions and 3 and 6 month outcomes have been reported previously[6].

### Follow-up

We followed-up the original participants using a postal questionnaire (with two reminders) between 5-7 years later (participants had been recruited over a 3 year period, mean time to follow-up was 6 years).

### Outcome measures

The primary outcome was improvement in one or more symptoms of which cure (no symptoms) is a subset. This was assessed using validated symptom severity questions, comprising the symptoms of incontinence, urgency, frequency and nocturia, obtained by postal questionnaire[7]. The questions were originally validated for use in an interviewer assisted questionnaire. In order to assess their use in a postal questionnaire we undertook a validation study in a sample of 85 participants stratified by age and gender. Kappa statistic was used to compare the interview and postal questionnaire responses for each question. Overall agreement was good. Most kappa statistics were over the threshold value of 0.5 indicating 'moderate' agreement (the majority were 0.7). The secondary outcome measures are divided into two groups: original secondary outcomes, i.e. those which were recorded at 3 and 6 months and again at long term follow-up, and additional secondary outcomes, i.e. those that were collected in order to supplement the original data and take account of important changes e.g. with regard to symptom sub groups. Original secondary outcome measures included:, a) Leicester impact scale, a validated instrument measuring impact on quality of life[8] (a scale developed for the study - with a range of 0 to 42 (there were 21 items with a maximum score of 2 on each item); b) patient perception of problem (Question: How much of a problem would you say you have with your urinary symptoms?) and satisfaction with current symptoms (Question: If you had to spend the rest of your life with urinary symptoms as they are now, how would you feel? Would you be....); and c) costs. Additional secondary outcomes included: d) outcomes according to symptom subgroups; e) predictors of long term improvement; and f) health related quality of life measured using the EQ5 D at follow-up. These outcomes were identified to determine whether there were any specific predictors of improvement, for example symptom subgroups that would enable service providers to target services of the future to specific groups.

### Statistical analysis

Effectiveness of the intervention at long term follow-up was analysed by 'intention to treat'. Results were expressed as absolute differences between observed proportion of individuals with each symptom in the two groups and proportions of individuals who improved (together with corresponding 95% confidence interval [CI]). Satisfaction with services was reported descriptively. A χ^2 ^test was used to test the difference in proportions and a *t*-test on the absolute difference in impact score from baseline was used for analysing impact score. In order to identify whether there were any identifiable baseline predictors of improvement at follow-up (in terms of number of symptoms), multivariate logistic regression was used to both simultaneously assess the predictive effect of a variety of factors and also to explore the evidence of any treatment-covariate interactions. The 2728 patients with follow-up data at 6 years gave the power to detect a minimum clinically significant difference of 10% (from 50-60%) at the 1% significance level with over 95% power as the original trial had been designed specifically to adequately power a number of small embedded trials.

### Costing Study

The original trial estimated the cost-effectiveness of the nurse led service in terms of cost per symptom alleviated and cost per 'case cured'. To examine how resource usage and hence costs have changed we included questions for resource use in the long-term follow up survey using similar methodology to the original trial. These covered: padding, aids and appliances, contacts with healthcare professionals, and use of hospital services. These questions asked specifically about resource use and expenditure in the last six months. We were unable to ask about the whole 5-7 year period due to unreliable recall. The costs used in the original trial were based on 2000/1 UK£s. As these would not reflect current costs we updated the costs in this analysis to the cost year 2006/7 using relevant recent sources of unit costs [9-11]. Where costs had been calculated from data obtained from the earlier trial we updated using the retail price index[12]. Analysis of missing data indicated that cases with missing data were statistically significantly different, and worse, in terms of health and urinary symptoms (the extent of missing data in the symptom and QOL data was minimal). For this reason we imputed missing data using regression-based multiple imputation implemented via the ICE macro in STATA version 10 [13].

Ethical Approval for this study was given by Leicestershire Local Research Ethics Committee Two (LREC reference:05/Q2502/9).

## Results

**Figure 1:**
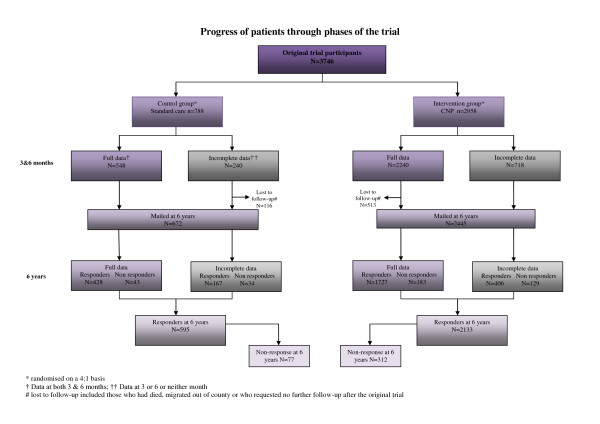
**Progress of patients through phases of the trial (separate file)**.

Of the 3746 individuals who took part in the original trial and therefore comprised our sample, 332 had died, 107 requested no further contact on completion of the original trial and 190 had migrated out of county, of the 3117 remaining a response rate of 87% (n = 2728) was achieved following 3 mailings (Figure 1. flow chart). There were no significant baseline differences in those originally recruited (n = 3746) and those who were followed up (n = 2728) in terms of age, gender, ethnicity, long term health or urinary symptoms (Table 1). The randomised groups were also similar. In addition we looked at baseline differences between responders at follow-up, non-responders at follow-up (n = 389), those who had died since the original trial (n = 332), those who had migrated out of county (n = 190) and those who requested after the original trial that they did not wish to be mailed again (n = 107). Table 1 shows that each of the groups were broadly similar at baseline in terms of demographics and urinary symptoms with the exception of those who died, these individuals were older and more likely to be male than other responders/non-responders recorded. The mean length of follow-up from baseline was 6 years.

**Table 1:** Baseline demographics and clinical characteristics of follow-up participants and non-responders to follow-up.

	RESPONDERS		NON- RESPONDERS			
	Baseline characteristics of responders at 6 years	Baseline characteristics of responders at 6 years	Non-responders to follow-up	Do not mail	Migrants	Deaths
	Intervention	Standard care				
Number of patients	2133	595	389	107	190	332
**Age group in years**						
**40-49 (%)**	429 (20%)	125 (21%)	110 (29%)	14 (13%)	49 (26%)	7 (2%)
**50-59 (%)**	644 (30%)	164 (27%)	110 (29%)	40 (37%)	61 (32%)	25 (7%)
**60-69 (%)**	638 (30%)	183 (31%)	72 (19%)	37 (35%)	46 (24%)	70 (21%)
**70-79 (%)**	368 (17%)	106 (18%)	69 (18%)	13 (12%)	27 (14%)	139 (42%)
**> 80 (%)**	54 (3%)	17 (3%)	28 (7%)	3 (3%)	7 (4%)	91 (27%)

**Female (%)**	1351 (63%)	360 (60%)	259 (67%)	70 (65%)	129 (68%)	130 (39%)
**Ethnic group**						
**white (%)**	2029 (95%)	561 (94%)	346 (89%)	92 (86%)	181 (95%)	324 (98%)
**Long-term illness (%)**	773 (36%)	211 (35%)	149 (38%)	43 (40%)	58 (31%)	187 (56%)

***Symptoms***						
**Leakage**	1713 (80%)	470 (79%)	324 (83%)	89 (83%)	158 (83%)	256 (77%)
**Frequency**	1121 (52%)	280 (47%)	219 (56%)	49 (46%)	99 (52%)	171 (51%)
**Urgency**	1359 (64%)	401 (67%)	262 (67%)	67 (63%)	115 (60%)	252 (76%)
**Nocturia**	714 (33%)	205 (34%)	144 (37%)	35 (33%)	66 (35%)	191 (57%)

**Mild or no problem**	1158 (54%)	326 (55%)	190 (49%)	63 (59%)	101 (53%)	149 (45%)
**Impact-Median (IQR)**	5 (2, 10)	4 (2, 9)	6 (2, 11)	5 (2, 11)	5 (3, 11)	4 (1, 10)
**Satisfied with current urinary symptoms for rest of life**	666 (31%)	212 (36%)	92 (24%)	26 (24%)	52 (27%)	110 (33%)

### Primary Outcome

#### Improvement/cure in urinary symptoms

Overall, at long-term follow-up significantly more individuals in the CNP group (72%) had improved (had fewer symptoms) compared with 67% in the SC group (difference of 5% (95% CI = 0.6 to 9;p = 0.02)). At 6 years the proportion reporting no symptoms or 'cured' was 31% in the intervention group and 27% in the SC group (difference of 4%, (95% CI -0.4 to 8 p = 0.08)). Changes in individual urinary symptoms (leakage, frequency, urgency and nocturia) were not significantly different between the two groups at follow-up (see table 2) despite a significant difference having been observed at 3 and 6 month follow-ups.

**Table 2:** Number of individuals with each symptom and no symptoms at 3 months, 6 months and long term follow-up (6 years) by randomisation group.

		Intervention		Standard care		Difference (95% Cl; P value)
		Total responders	Individuals with symptoms/event (%)	Total responders	Individuals with symptoms/event (%)	
**Improvement**	**3-months**	2378	1417 (60%)	584	281 (48%)	11% (7 to 16; P = < 0.001)
	**6 months**	2201	1369 (62%)	536	277 (52%)	11% (6 to 15 P < 0.001)
	**Long term follow-up**	2045	1530(72%)	567	380 (67%)	5% (0.6 to 9; P = 0.02)

**No symptoms (cure)**	**3-months**	2378	591 **(25%)**	584	88 **(15%)**	10% (6% to 13%; p < 0.001)
	**6 months**	2201	624 (**28%**)	536	104 **(19%)**	9% (5% to 13%; p < 0.001)
	**Long term follow-up**	2069	643 (31%)	571	156 (27%)	4% (-0.4 to 8; P = 0.08)

**Leakage** (several times per month or more)	**Baseline**	2958	2392 (**82**%)	788	618 (**79**%)	
	**3 months**	2483	1567 (**63%**)	612	428 (**70%**)	-7% (-11% to -3%; p = 0.002)
	**6 months**	2235	1362 (**61%**)	546	356 (**65%**)	-4% (-9% to 0%; p = 0.066)
	**Long term follow-up**	2093	1166 (**56**%)	580	334 (**58**%)	-2% (-6 to 3; P = 0.4)
						

**Frequency** (hourly or more)	**Baseline**		1563 (53%)		376 (48%)	
	**3 months**	2428	723 (**30%**)	598	219 (**37%**)	-7% (-11% to -2%; p = 0.001)
	**6 months**	2231	539 (**24%**)	545	182 (**33%**)	-9% (-14% to -5%; p < 0.001)
	**Long term follow-up**	2098	290 (14%)	576	85 (15%)	-1% (-4 to 2; P = 0.6)
						

**Urgency** (very strong or overwhelming)	**Baseline**		1927 (65%)		529 (67%)	
	**3 months**	2503	819 (**33%**)	618	248 (**40%**)	-7% (-12% to -3%; p = 0.001)
	**6 months**	2236	682 (**31%**)	546	228 (**42%**)	-11% (-16% to -7%; p < 0.001)
	**Long term follow-up**	2108	482 (23%)	583	153 (26%)	-3% (-7% to 0.6%;p = 0.09)
						

**Nocturia** (3 times per night or more)	**Baseline**		1070 (36%)		285 (36%)	
	**3 months**	2502	497 (**20%**)	617	164 (**27%**)	-7% (-11% to -3%; p < 0.001)
	**6 months**	2236	420 (**19%**)	547	133 (**24%**)	-6% (-9% to -2%; p = 0.004)
	**Long term follow-up**	2106	437 (21%)	585	135 (23%)	-2% (-6 to 2; P = 0.2)

#### Original Secondary Outcomes

##### a) Impact on activities and feelings (QoL)

Absolute change from baseline on the overall quality of life scale was calculated at follow-up to be 4 (Inter Quartile Range IQR 1-9) in the CNP group and 4 (IQR 1 to 10) in the SC group. The mean difference (between CNP and SC) in change from baseline was 0.31 (95% CI: -0.46 to 1.08), P = 0.4

##### b) Perception of problem and satisfaction with symptoms

By long term follow up differences between the CNP and SC groups in terms of reported 'no problem' or 'mild problem' and satisfaction with current symptoms had diminished. Although in each case the CNP arm gave more positive responses there was no significant differences between the groups (Table 3).

**Table 3:** Patient perception of problem and satisfaction with current urinary symptoms at 3 and 6 months and 6 years presented by randomisation group.

	**Continence Nurse Practitioner**		**Standard Care**		
			
	**Total number of individuals**	**Number of individuals with event**	**Total number of individuals**	**Number of individuals with event**	**Difference (95% CI; p-value)**
	
	**Mild or no problem**				
**3 months**	2468	819 (74%)	614	416 (68%)	6% (2% to 10%; p = 0.003)
**6 months**	2181	1721 (79%)	545	380 (70%)	9% (5% to 13%; p < 0.001)
**Long term follow-up (6 Years)**	2104	1474 (70%)	589	406 (69%)	1% (-3 to 5%;p = 0.6)
	**Satisfied with current urinary symptoms for rest of life**				
					
**3 months**	2498	1294 (52%)	618	276 (45%)	7% (3% to 12%; p = 0.001)
**6 months**	2236	1428 (64%)	546	289 (53%)	11% (6% to 16%; p < 0.001)
**Long term follow-up (6 Years)**	2109	1152 (55%)	591	306 (52%)	3% (-2 to 7%; P = 0.2)

##### c) Results of the costing study

The results of the costing study from the follow up survey are presented in Table 4. We had complete cost data for 2217 out of 2728 cases.

**Table 4:** Summary of costs collected retrospectively over the 6-month period prior to the follow-up study

		CNP	SC	CNP v SC
			
MALE	Cost component	Mean [SE] (95% CI)	Mean [SE] (95% CI)	P-value
	Total NHS (excl. P)	91.72 [45.00] (3.42,180.02)	61.61 [32.36] (-1.90,125.11)	0.2
	Total Own Borne	2.25 [5.09] (-7.73,12.23)	3.89 [4.40] (-4.75,12.53)	0.6
	Total NHS + Own (excl. P)	93.97 [48.17] (-0.55,188.49)	65.50 [36.43] (-5.99,136.99)	0.3
FEMALE	Cost component	Mean [SE] (95% CI)	Mean [SE] (95% CI)	P-value
	Total NHS	84.96 [28.29] (29.48,140.44)	60.00 [10.40] (39.61,80.39)	0.2
	Total Own Borne	29.22 [6.63] (16.22,42.21)	27.67 [5.86] (16.18,39.16)	0.6
	Total NHS + Own	114.18 [29.18] (56.95,171.40)	87.67 [12.36] (63.44,111.91)	0.2

Updated cost analysis using multiple imputation to deal with missing data (covariates used to impute: 4 urinary symptoms (urgency, leakage, nocturia and frequency), number of symptoms, age, body mass index & EQ-5D).

There were no statistically significant differences in costs between the CNP and the GP groups. NHS costs for both men and women were higher in the CNP group. However, NHS costs for both groups were similar for men and women. Costs borne by respondents themselves were higher for women (due to much higher spending on pads). Total costs were substantially higher for the CNP group, due to higher NHS costs in both males and females.

#### Additional Secondary Outcomes

##### *d) *Outcomes according to symptom sub group

Sub -group analysis was undertaken to determine whether outcomes were different for specific symptom sub groups. Those identified were the International Continence Society (ICS) defined symptom sub-groups of Urodynamic Stress Incontinence (USI), Overactive bladder (OAB) & Mixed symptoms [14]. Table 5 displays results at long-term follow-up in terms of improvement (i.e. reduction in the number of symptoms present compared to baseline) and 'cure' (i.e. no symptoms present at follow-up) between the CNP and standard care arms of the trial stratified by ICS classification (USI, OAB and Mixed) at baseline (i.e. randomisation). Only for those patients who received an initial classification of Mixed did the use of a CNP appear to be statistically significantly better than standard care (P = 0.01). The category 'other' comprises those without an ICS classification and included those with individual symptoms of nocturia, frequency or continuous leakage.

**Table 5:** Baseline classification and outcome at long-term follow-up

	CNP		SC		Difference (95% CI)	*P-value for Interaction*
	
	Total	Events (%)	Total	Events (%)		
Improvement						
USI	233	158 (68)	56	35 (63)	5% (-9 to 19)	
OAB	358	254 (71)	123	96 (78)	-7% (16 to 2)	*0.05*
Mixed Other *	1109 315	832 (75) 202 (64)	297 84	200 (67) 46 (55)	8% (2 to 14) 9% (-3 to 21)	
Cure						
USI	235	105 (45)	56	19 (34)	11% (-3 to 24)	
OAB	363	94 (26)	124	40 (32)	-6% (-16 to 3)	*0.06*
Mixed Other *	1120 316	261 (23) 165 (52)	300 84	63 (21) 33 (39)	2% (-3 to 8) 13% (1 to 25)	

*Includes those with nocturia, frequency or continuous leakage

##### *e) *Predictors of Long-term Improvement

Those patients randomised into the CNP arm had a 26% (95% CI: 4% to 54%) increased relative odds of improving compared to the SC arm [Odds Ratio 1.26, 95% CI: 1.04 to 1.54; P = 0.02]. After adjustment for age, gender and baseline ICS classification there was little change in terms of the estimate of effect of CNP compared to SC [OR 1.25, 95% CI: 1.02 to 1.53, P = 0.03]. Of the other factors considered only the presence of an initial classification of Mixed appeared to have any statistically significant relationship with outcome (i.e. improvement) though there was little evidence of an interaction between treatment and ICS classification (P = 0.05). Odds ratios for improvement were as follows; Mixed *cf*. OAB 1.07 (95% CI 0.81 to 1.42; p = 0.6), Mixed *cf*. USI 1.41 (95% CI 1.07 to 1.85; p = 0.02) and OAB *cf*. USI 1.31 (95% CI 0.91 to 1.88; p = 0.1) the 'mixed' effect is likely to be due to OAB improvement.

##### *f) *EQ-5 D at Follow-up

Table 6 shows the results of the trial in terms of the health related quality of life instrument EQ-5 D [15] (Euroqol) at follow-up split according to treatment arm and whether patients improved, remained the same or worsened at follow-up compared to baseline. There were no overall differences in EQ-5 D between the two treatment arms. Although there were no overall differences in terms of EQ-5 D between the two treatment arms, those patients who improved, i.e. had fewer or no symptoms at follow-up compared to baseline, had statistically higher EQ-5 D scores than those who did not, and this was seen in both treatment arms, although there was between patients who had improved or worsened -0.0036 (-0.06-0.012, 0005) regardless of trial group.

**Table 6:** EQ-5 D at follow-up according to treatment arm and improvement at follow-up.

	**CNP**		**SC**		**Overall**	
	
	**n**	**Mean (SE)**	**n**	**Mean (SE)**	**n**	**Mean (SE)**
	
Improved	1441	0.720 (0.007)	370	0.724 (0.014)	1811	0.721 (0.010)
Same	436	0.705 (0.014)	150	0.716 (0.022)	736	0.685 (0.011)
Worse	118	0.611 (0.029)	32	0.536 (0.056)		
						
**Overall**	2074	0.710 (0.006)	577	0.701 (0.012)		
						
Difference (95% CI & P-value)	0.008 (-0.018 to 0.034; 0.5)				-0.036 (-0.060 to -0.012; 0.005)	

## Discussion

### Summary of main findings

**Figure 2:**
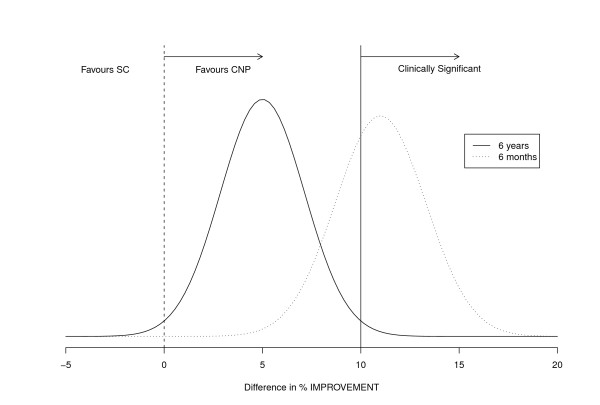
**Differences in percentage improvement at 6 months and 6 years in the CNP and standard care arms (separate file)**.

In the original trial we had sufficient power to detect a clinically significant minimum difference in the proportion of individuals who improved their symptoms of 10% to be detected at the 1% significance level with 99% power. At 6 month follow-up we detected a difference in improvement of 11% which met the criteria for clinical significance. By long term follow up we detected a 5% difference which whilst a statistically significant improvement did not reach our threshold for clinical significance. So, at 6 years, whilst the probability of SC being superior is less than 1%, so too is the probability of the difference being greater than 10% (compared with 67% at 6 months (Figure 2).

The improvements in the CNP group shown immediately post treatment (at 3 and 6 months) had decreased (by half) at long term follow up. The follow-up took place at 6 years therefore the annual 'drop off' of effect between the groups was approximately 1% each year.

### Comparison with existing literature

Although this trial has shown no clinically significant difference between treatment groups at 6 years, the statistically significant difference of 5% is an improvement on previous comparable trials which have shown no difference at all at 6 years. Maintenance of effect is rarely seen in long term follow-up studies of conservative interventions for incontinence [16]. Glazener et al 2005 [17] in their 6 year follow-up of a randomised controlled trial of conservative management of postnatal urinary and faecal incontinence found no differences between their intervention and standard care groups at 6 years following a 1 year difference of 9%. Similarly Agur et al (2008) [18] in their eight year follow-up of an RCT of antenatal pelvic floor muscle training found no difference between the groups at follow-up.

### Strengths and limitations of the study

This study gained a response rate of 87%, this was a high response rate compared to similar follow-ups at six years [17].

Secondary outcomes of improvement in impact, perception of their problem and satisfaction with quality of life seen at 3 and 6 months were not maintained at long term follow-up.

For costs at follow-up our most notable finding was that NHS costs in the CNP arm were higher than those in the SC arm, though not significantly so. This finding was surprising given the lack of differences found in the other variables examined in the course of this study. However, this may be due to the fact that those in the CNP arm were familiar with seeking and obtaining specialist help with their urinary symptoms. A higher propensity to seek primary care contacts may in turn lead to more referrals to secondary care and more inpatient care and surgeries. There were no differences in individuals own borne costs suggesting that measures taken by individuals to manage their own symptoms were similar in the two groups. We found that NHS costs were similar for men and women. We were able to exclude many prostate related costs from our calculations as we could identify prostate related inpatient stays and drug usage from our estimates (including these would increase NHS costs from £92 to £123). Some of the costs related to male urinary symptoms would still be related to prostate conditions however as the reasons for services such as contact with GP may not be related to the underlying condition.

Since the 2002 publication of the ICS standardisation document [14] the symptom subgroups of USI, OAB and mixed have become more established and we wanted to explore whether long term outcomes varied according to these subgroups. If certain subgroups of individuals did better at long term follow-up than others, future services could specifically identify and target these groups for services in the future. We only found that those individuals initially classified with 'mixed' incontinence did statistically better in the CNP arm. This may be due to the nature of the service which was better able to provide more complex care tackling more than one problem and following up outcomes to amend treatment interventions in a way that standard care is less able to do. In addition mixed symptoms tend to be more severe and may have responded well to the comprehensive CNP service.

At long term follow-up there is the potential risk of contamination between treatment arms once a trial is finished. This was not the case in this study as the CNP service was specifically set up for study purposes and funding of the service was not possible following completion of the research programme. The availability of generic continence services post trial for each group was identical. Whilst these services would draw on the education and experience of nurses trained within the trial programme, there was no possibility that those from standard care would have access to the successful service and could be contaminated.

Although the study was not explicitly designed as a cluster randomised trial, 220 (8%) patients were in the same household, i.e. 110 household pairs. The reduction in power due to this cluster was minimal - the study was designed to have over 95% power in order to adequately power a number of small embedded trials. In order to assess any impact on the results, a logistic regression model for the primary outcome (improvement compared to baseline) and which allowed for the effect of clustering was used. This gave identical results to those presented.

We were also interested to know whether there were any specific predictors of improvement, that again would enable service providers to target services of the future to specific groups, however there were no clear predictors of improvement.

## Conclusions

This is the first study to examine the long term effectiveness of a nurse led service on storage symptoms. The modest effects shown at long term follow up indicate a drop-off of effect with time which is likely to be related to the fact that the interventions taught at the initial intervention needed to be continued or periodically reinforced e.g. pelvic floor exercises and bladder training. By implementing the initial interventions and then offering periodic 'top-ups' in the form of nurse contacts with patients to reinforce teaching on bladder training, fluid and diet advice and pelvic floor exercises, a lasting effect may be achieved. Such suggestions would need to be rigorously tested as part of a RCT with a full economic evaluation, following exploratory work on the potential components of the proposed 'top-ups'.

## Competing interests

The authors declare that they have no competing interests.

## Authors' contributions

KSW, KA, CMcG, RPA, DT, NC, and CS contributed to the design of the study, and KSW supervised the execution of the study and data collection. DC co-ordinated and undertook data collection and data entry. KRA, NC, DC, KSW, DAT, analysed the data. All authors were actively involved in checking the data and critical revisions to the manuscript, which was drafted by all authors.

## Pre-publication history

The pre-publication history for this paper can be accessed here:

http://www.biomedcentral.com/1472-6963/11/58/prepub
